# Genetic Dissection of a QTL Affecting Bone Geometry

**DOI:** 10.1534/g3.116.037424

**Published:** 2017-01-11

**Authors:** Olivia L. Sabik, Juan F. Medrano, Charles R. Farber

**Affiliations:** * Center for Public Health Genomics, School of Medicine, University of Virginia, Charlottesville, Virginia 22908; † Department of Biochemistry and Molecular Genetics, School of Medicine, University of Virginia, Charlottesville, Virginia 22908; ‡ Department of Animal Science, University of California, Davis, California 95616; § Department of Public Health Science, School of Medicine, University of Virginia, Charlottesville, Virginia 22908

**Keywords:** quantitative trait loci (QTL), bone geometry, bone strength, QTL fine-mapping, allele-specific expression (ASE)

## Abstract

Parameters of bone geometry such as width, length, and cross-sectional area are major determinants of bone strength. Although these traits are highly heritable, few genes influencing bone geometry have been identified. Here, we dissect a major quantitative trait locus (QTL) influencing femur size. This QTL was originally identified in an F2 cross between the C57BL/6J-hg/hg (HG) and CAST/EiJ strains and was referred to as femur length in high growth mice 2 (*Feml2*). *Feml2* was located on chromosome (Chr.) 9 at ∼20 cM. Here, we show that the HG.CAST-(*D9Mit249-D9Mit133*)/Ucd congenic strain captures *Feml2*. In an F2 congenic cross, we fine-mapped the location of *Feml2* to an ∼6 Mbp region extending from 57.3 to 63.3 Mbp on Chr. 9. We have identified candidates by mining the complete genome sequence of CAST/EiJ and through allele-specific expression (ASE) analysis of growth plates in C57BL/6J × CAST/EiJ F1 hybrids. Interestingly, we also find that the refined location of *Feml2* overlaps a cluster of six independent genome-wide associations for human height. This work provides the foundation for the identification of novel genes affecting bone geometry.

Osteoporosis is a disease of severe bone loss that leads to skeletal fragility and an increased risk of fracture (Black and Rosen 2016). In the U.S., osteoporosis affects over 12 million people and is directly responsible for 1.5 million fractures annually (Black and Rosen 2016). Although fracture is not commonly associated with mortality, of the ∼300,000 people each year that suffer a hip fracture, one in five will die in the subsequent 12 months (Gass and Dawson-Hughes 2006).

Bone geometry is one of the many factors that contribute to bone strength (Bouxsein and Karasik 2006). Studies in mice have demonstrated that up to 50% of the variance in bone strength is due to bone size (Ng *et al.* 2007). Furthermore, the relationship between bone geometry and fracture risk in humans has been demonstrated in both the wrist and the spine, as decreased cross-sectional area of the radius and vertebrae are associated with increased risk of fracture (Bouxsein and Karasik 2006). In addition, similar to most other characteristics of bone, bone geometry is highly heritable (*h*^2^ > 0.50) and amenable to genetic analysis (Norgard *et al.* 2008). Therefore, increasing our understanding of the genes influencing bone geometry using genetic analyses has the potential to inform strategies for the treatment and prevention of bone fragility.

To identify QTL affecting body composition, Corva *et al.* (2001) generated an F2 cross between the C57BL/6J-*hg/hg* (HG) and CAST/EiJ (CAST) strains. The HG strain is a C57BL/6J (B6) mouse that is homozygous for a deletion encompassing the *Socs2* gene [the high growth (*hg*) locus], a negative regulator of growth hormone signaling, that results in increased growth and body size (Metcalf *et al.* 2000; Horvat and Medrano 2001). In contrast, CAST mice are genetically divergent wild-derived inbred mice that are small and lean. One QTL identified in the *hg* × CAST cross was *Feml2*. *Feml2* was located on Chr. 9 at ∼20 cM, explained 10.7% of the variance in femur length, and was independent of the *hg* locus (Corva *et al.* 2001). In order to identify the gene(s) responsible for *Feml2*, we generated the HG.CAST-(*D9Mit249-D9Mit133*)/Ucd (HG9) congenic strain that possessed CAST alleles from 9 to 84 Mbp on an HG background (Farber *et al.* 2006). The HG9 strain has previously been used to fine-map a distinct QTL affecting adiposity (Farber *et al.* 2009).

In the current study, two HG9 F2 intercrosses were used to fine-map *Feml2*. We used the complete genome sequences of the B6 and CAST strains and ASE analysis of B6 × CAST F1 mice to identify candidate genes driving the effect on femur length. Interestingly, the human syntenic region contains a cluster of six genome-wide associations with human height. These data provide the foundation to identify genes contributing to bone size and geometry.

## Materials and Methods

### Mouse strains and husbandry

The mouse strains used in the study were B6, HG, and the HG9 (MGI:3771219) congenic strain. The development of the HG and HG9 congenic strains have been previously described (Horvat and Medrano 1995; Farber *et al.* 2006). B6 and HG mice were obtained from vivarium stock. Mice were provided with a normal chow diet (Purina 5008; 23.5% protein and 6.5% fat, 3.3 Kcal/g) and water *ad libitum*, and housed in groups of 2–5 in polycarbonate cages bedded with a 2:1 mixture of CareFRESH (Absorption, Ferndale, WA) and soft paper chips (Canbrands, Moncton, Canada). Mice were maintained under controlled conditions of temperature (21 ± 2°), humidity (40–70%), and lighting (14 hr light, 10 hr dark, lights on at 7 am). All mouse protocols were managed according to the guidelines of the American Association for Accreditation of Laboratory Animal Care (AAALAC) and approved by the Institutional Animal Care and Use Committee at the University of California, Davis.

### Characterization of bone geometry in HG9 congenic mice

At 9 wk of age (± 5 d), male HG9 congenic (*N* = 7) and HG control (*N* = 14) mice were anesthetized and body weights and lengths were measured to the nearest decigram and centimeter, respectively. Mice were then killed and femurs were removed and cleaned of soft tissue. For each femur, we measured the length and width, in the mediolateral and anterior–posterior orientations, using digital calipers (Mitutoyo Corporation, Takatsu-ku, Japan). Experimenters were blinded to the genotype of the mice.

### Development and characterization of the HG9F2 mapping populations

The congenic F2 mouse populations used in this study have been described in (Farber *et al.* 2009). Briefly, HG9 × HG (*N* = 283) and HG9 × B6 (*N* = 457) male and female F2 mice were generated by intercrossing F1 mice. F2 mice were phenotyped as described above for HG9 congenics. Mice were genotyped using microsatellite markers. HG9 × B6 F2 mice were genotyped for the *hg* locus as described in Farber *et al.* (2006).

### Feml2 fine-mapping

All statistical analyses were performed using the R Language and Environment for Statistical Computing (R Development Core Team 2013). The R/qtl package was used to perform the linkage analysis (Broman *et al.* 2003). Sex-averaged genetic maps were generated and conditional genotype probabilities were estimated using the calc.genoprob function, along the length of the congenic donor region at 0.1 cM intervals. Both F2 crosses were combined for the linkage analysis. The scanone function, using the Haley–Knott regression algorithm, was used to perform interval mapping using a model that included sex, body weight, and cross type (HG9 × HG = 1 or HG9 × B6 = 2) terms as additive covariates. LOD significance for all models tested were empirically determined using 1000 permutations. We converted genetic to physical distance by regressing megabase pairs onto centimorgans for all markers.

### Growth plate RNA collection

Seven male F1s from a cross between B6 and CAST were killed at 21 d of age by isoflurane anesthesia followed by cervical dislocation. Proximal and distal tibial growth plates were rapidly dissected, placed in TRIzol (Ambion by Life Technologies) and pulverized using the Tissue Tearor homogenizer (BioSpec Products). Total RNA was extracted from homogenized tissue (mirVana miRNA Isolation Kit, Ambion by Life Technologies). RNA concentration was measured by fluorometry (Qubit 2.0 Fluorometer, Life Technologies).

### RNA-sequencing (RNA-seq) sample preparation

RNA-seq libraries were constructed from 200 ng of total RNA using Illumina TruSeq Stranded Total RNA with Ribo-Zero Gold sample prep kits (Illumina, Carlsbad, CA). Constructed libraries contained RNAs > 200 nucleotides (both unpolyadenylated and polyadenylated) and were depleted of cytoplasmic and mitochondrial rRNAs. An average of 6.7 million 2 × 75 bp paired-end reads were generated for each sample on an Illumina NextSequation 500 (Illumina).

### RNA-seq alignment strategy and ASE analysis

Alleles was generated sing g2gtools (Choi 2016a), a transcriptome containing both B6 (mm 10) and CAST [mm10 with version 4 single nucleotide polymorphisms (SNPs) and insertion/deletions (indels) from the Mouse Genome Project http://www.sanger.ac.uk/science/data/mouse-genomes-project]. Reads from each F1 hybrid were aligned to the joint transcriptome using Bowtie, allowing no more than 3 mismatches and reporting all alignments of the stratum containing the fewest number of mismatches (Langmead *et al.* 2009). Next, EMASE (Choi 2016b) was used to quantify the number of reads from both the maternal and paternal allele. Each end of the paired-end samples was processed separately and only reads aligning in both samples were included. Similarly, each lane was processed separately in order to correct for lane-specific effects. Postquantification, all samples passed quality control checks based on the expected global proportions of reads aligning to each parental strain. All samples showed nearly equal reads mapping to each parental strain. In order to identify genes showing allelic expression, edgeR was used to compare the quantity of each transcript by strain across the seven samples. Only those transcripts with measured expression in at least one haplotype in all of the samples were included in ASE analysis. The glmFit and glmLRT functions were used to statistically compare the expression of each transcript between the CAST and B6 alleles.

### Additional statistical analyses

Bone geometry measures in HG9 congenic and HG control mice were compared using a Student’s *t*-test in R (R Development Core Team 2013). Comparisons at a *P* < 0.05 were deemed significant. Permutation analysis was used to determine the probability of six GWAS associations for height occurring within the 7.5 Mbp human region syntenic with *Feml2* by randomly selecting 1000 7.5 Mbp regions from the genome and counting the number of associations within each region. Genome-wide significant SNPs identified through a GWAS for human height were mapped to mm10 from hg18 using the liftover tool from UCSC.

### Data availability

Both C57BL/6J and CAST/EiJ strains are commercially available from Jackson Labs. Supplemental Material, File S1 contains R/QTL formatted mapping information, including mouse IDs, phenotype information, and SNP marker identifiers, locations, and genotypes (A=C57BL/6J, B=CAST/EiJ) (File S1). Phenotype information can be found in the accompanying README file (File S2). Gene expression data from chondrocytes are available at GEO with the accession number GSE90055.

## Results

### Feml2 is captured in HG9 mice

We characterized femur geometry in HG9 and HG male mice. As shown in Table 1, femur length was decreased by 6% (*P* = 1.3 × 10^−5^) in HG9 males, consistent with the effects of *Feml2* in the original F2 cross (Corva *et al.* 2001). In addition, mediolateral and anterior–posterior femur widths were also decreased by 6–7% (*P* = 2.1 × 10^−3^ and *P* = 3.7 × 10^−2^, respectively) in HG9 males (Table 1). These data confirm the capture of *Feml2* in the HG9 congenic interval.

**Table 1:** Characterization of femur geometry in male HG and HG9 mice

	HG (*N* = 14)	HG9 (*N* = 7)	Difference (%) (HG9 – HG/HG)	*P*-Value
Femur length (mm)	17.2 ± 0.1	16.2 ± 0.1	−6	1.3 × 10^−5^
Mediolateral femur width (mm)	2.41 ± 0.04	2.28 ± 0.02	−6	2.1 × 10^−3^
Anterior–posterior femur width (mm)	1.55 ± 0.05	1.45 ± 0.02	−7	3.7 × 10^−2^

### High-resolution mapping of Feml2

**Figure 1:**
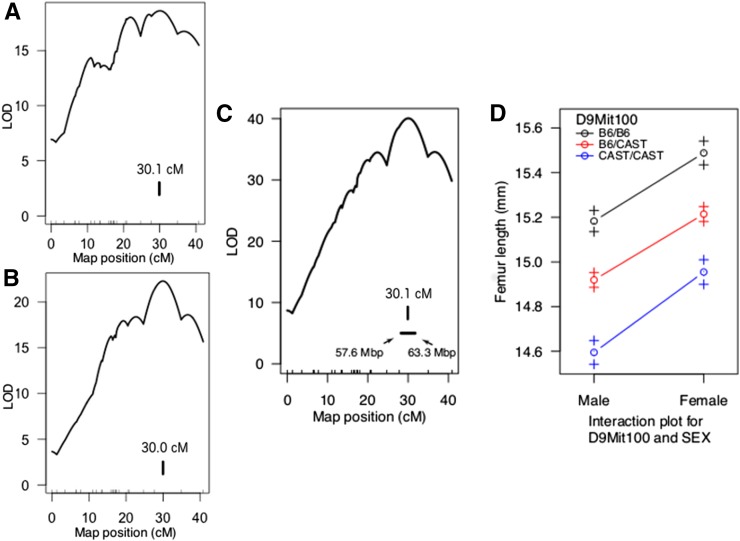
*Feml2* LOD score profiles. Vertical lines indicate peak LOD scores. The peak of *Feml2* was located at 30.1 cM in the HG9 × HG (*N* = 283) cross (A), 30.0 cM in the HG9 × B6 (*N* = 457) cross (B), and 30.1 cM in both crosses combined (*N* = 740). The 95% C.I. for the location of *Feml2* in the combined cross was 28.3–31.7 cM (57.6–63.3 Mbp) (C). *Feml2* had highly significant effects (*P* < 0.001) on femur length in both male and female mice (D). LOD, logarithm of the odds.

To refine the location of *Feml2* within the HG9 congenic interval, we generated two F2 crosses, HG9 × HG (*N* = 283) and HG9 × B6 (*N* = 457). Femur length was first mapped in each cross separately. No differences in the LOD score profile of peak positions were observed (Figure 1, A and B). As a result, both crosses were combined to increase mapping resolution (Figure 1C). *Feml2* mapped to 30.1 cM with a peak LOD score of 40.0 (Figure 1C). The effects of *Feml2* on femur length were additive with each CAST allele decreasing femur length by 0.23 mm (Figure 1D). *Feml2* explained 7.8% of the total variance in femur length. The 95% C.I. for *Feml2* extended from 28.0 to 31.7 cM, which in physical distance equated to the 5.7 Mbp region extending from 57.6 to 63.3 Mbp (GRCm38/mm10).

### Characterizing Feml2 variants between CAST and B6

*Feml2* contains a total of 69 RefSeq protein-coding genes (Table S1). Based on the sequenced CAST genome (Keane *et al.* 2011), *Feml2* contains 46,624 high-confidence SNPs between CAST and B6. Most (45,664; 97.9%) of the SNPs are noncoding (Table 2). There are 960 (2.1%) coding variants, of which 86 are potentially “high-impact.” Of these 86, there are 81 missense, one initiator codon, three stop-gained, and one stop-retained variants (Table 2). Potentially high-impact variants were found in 37 of the 69 *Feml2* genes. The initiator codon variant is in the enhancer of mRNA decapping 3 (*Edc3)* gene; however, a second in-frame “ATG” is located 6 bp downstream. Two of the stop-gain and the stop-retained variants were found in the “unclassified” gene *1700036A12Rik*. The other stop-gain variant was in another “unclassified” gene, *Gm10657* (Table S1).

**Table 2:** List of SNPs located within *Feml2*

Variant	Number
Noncoding	
Downstream gene variant	1993
Upstream gene variant	2230
Intergenic variant	20,949
Intron variant	20,452
Splice region variant	40
Total	45,664
Coding	
3′-UTR variant	523
5′-UTR variant	51
Synonymous variant	246
Missense variant	81
Initiator codon variant	1
Stop gained	3
Stop retained	1
Mature miRNA variant	1
Noncoding exon variant	53
Total	960

UTR, untranslated region; miRNA, microRNA.

In addition to SNPs, there were 8803 small indels. There were 189 indels in untranslated regions (UTRs), two frameshifts, and one in-frame insertion. The rest were intergenic. The two frameshift variants were found in *Arid3b* and *Nptn*; however, both occurred in exons predicted by Ensembl that were not part of the RefSeq transcript for either gene. The in-frame insertion was also found in the *Arid3b* gene and did occur with a RefSeq exon.

### Characterizing Feml2 ASE using CAST × B6 F1 RNA-seq data

**Figure 2:**
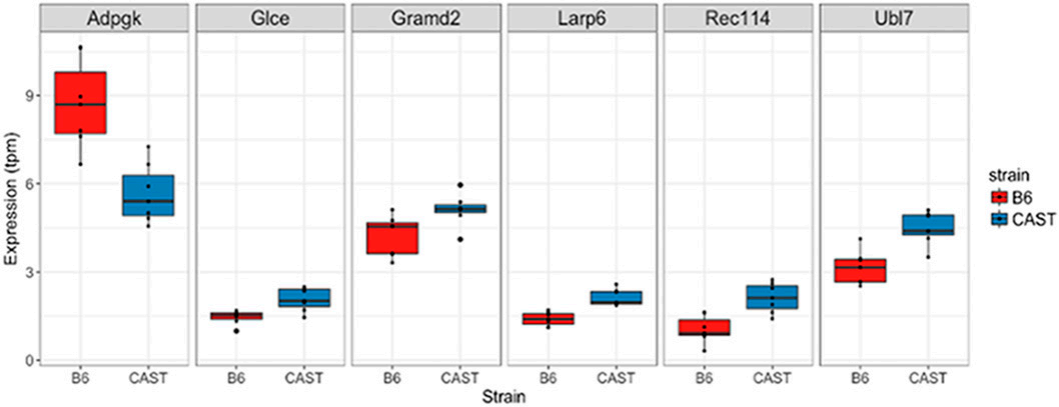
Boxplots of the expression of six genes demonstrating significant (FDR < 0.20) allele-specific expression differences. Expression is expressed in units of transcripts per million (tpm) and binned by strain of origin. FDR, false discovery rate.

**Figure 3:**
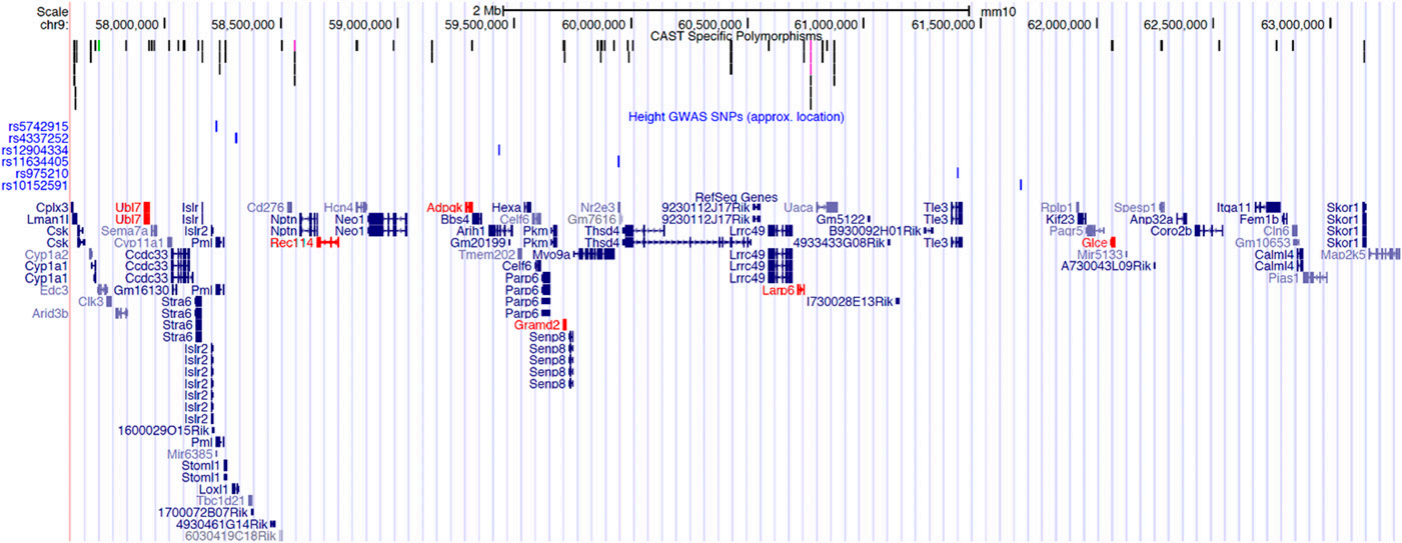
UCSC Genome Browser view of mm10 Chr. 9:57300000–63300000, which comprises the *Feml2* locus. The uppermost track displays potential high-impact predicted coding polymorphisms between CAST and B6 (black = missense, green = initiator codon variant, and purple = stop gained and stop retained variants). The second uppermost track (blue) contains SNPs homologous to those identified in a GWAS for human height (Wood *et al.* 2014), which may play a regulatory role in gene expression that influences femur length. Finally, the bottom-most track displays the genes within the *Feml2* region. In red are genes that exhibited significant (FDR < 0.20) differences in allele-specific expression. FDR, false discovery rate; GWAS, genome-wide association study; SNP, single nucleotide polymorphism; UCSC, University of California, Santa Cruz.

To identify *Feml2* genes whose expression is under genetic regulation, we quantified ASE in growth plate tissue in CAST × B6 F1 mice. Six of the 69 genes from the *Feml2* region were found to be expressed in an allele-specific manner, demonstrating higher transcript levels originating from either the B6 or CAST chromosomes in growth plate samples at an FDR < 0.20 (Figure 3 and Table 3). These genes are GRAM domain containing 2 (*Gramd2*), La Ribonucleoprotein Domain Family Member 6 or Acheron (*Larp6*), ADP-Dependent Glucokinase (*Adpgk*), Bone Marrow Stromal Cell-Derived Ubiquitin-Like 7(*Ubl7*), Meiotic Recombination Protein REC114-Like (*Rec114*), and Heparin/Heparan Sulfate:Glucuronic Acid C5-Epimerase (*Glce*). All of these genes, except for Adpgk, were preferentially expressed from the CAST allele as compared to the B6 allele (Figure 2).

**Table 3:** Allele-specifically expressed genes from the *Feml2* region

Gene Name	Chr.	Gene Start–End	CAST/EiJ Mean tpm	C57BL/6J Mean tpm	CAST/EiJ Mean Eff. Counts	C57BL/6J Mean Eff. Counts	logFC*^a^*	*P*-Value	FDR
Gramd2	9	59,680,144–59,718,874	5.11	4.22	534	339	0.69	0.002	0.11
Larp6	9	60,712,989–60,738,801	2.13	1.40	216	142	0.64	0.005	0.11
Adpgk	9	59,291,572–59,316,199	5.66	8.72	586	893	−0.60	0.013	0.13
Ubl7	9	57,910,986–57,929,968	4.49	3.14	247	174	0.54	0.012	0.13
Rec114	9	58,652,850–58,743,964	2.12	1.04	43	19	1.09	0.008	0.13
Glce	9	62,057,248–62,122,655	2.06	1.46	341	254	0.49	0.021	0.17

Chr., chromosome; tpm, transcripts per million; Eff., effect; FDR, false discovery rate.

a Positive logFC values correspond to favored CAST/EiJ expression, while negative logFC values correspond to favored C57BL/6J expression.

### Feml2 overlaps with a cluster of human height genome-wide associations

Given the significant impact of *Feml2* on femur length, it is possible that *Feml2* harbors multiple independent variants impacting skeletal dimensions. To determine if there is evidence that *Feml2* represents a “hot-spot” of genes influencing long bone size, we analyzed the syntenic region of the human genome for human height associations (which are often driven by changes in skeletal dimensions) (Soranzo *et al.* 2009) identified by GWAS (Wood *et al.* 2014). *Feml2* is syntenic with human Chr. 15 from 67.5 to 75.5 Mbp. This region contains six independent associations as identified by GWAS for human height (Wood *et al.* 2014) (Figure 3). Through permutation, there was suggestive evidence that the human region syntenic with *Feml2* contained more associations for height than would be expected by chance (*P* = 0.06). Additionally, these SNPs were found to be near the human homologs of all six genes exhibiting allelic expression (Table 4).

**Table 4:** Human homologs of genes in *Feml2* with observed ASE in CAST/EiJ × C57BL/6J F1 mice and the lead height-associated SNPs nearest the human genes

Gene	Start (Mbp)*^a^*	End (Mbp)*^a^*	Nearest rs ID#	Chr.	SNP Coord. (Mbp)*^a^*	Dist. to TSS (Kbp)
Gramd2	72.159807	72.197785	rs12904334	15	72.550363	390.556
Larp6	70.829130	70.854159	rs975210	15	70.072012	757.118
Rec114	73.443158	73.560014	rs4337252	15	73.934423	491.265
Ubl7	74.445977	74.461182	rs5742915	15	74.044291	401.686
Adpgk	72.751369	72.783785	rs12904334	15	72.550363	201.006
Glce	69.160634	69.272199	rs10152591	15	69.755817	595.183

ID#, identifier number; Chr., chromosome; SNP Coord., single nucleotide polymorphism coordinate; Dist., distance; TSS, transcription start site.

a Human genome build hg38.

## Discussion

As a first step in identifying the gene(s) responsible for a QTL with a major effect on bone geometry, we developed a congenic strain containing the *Feml2* QTL and conducted an F2 intercross to refine the location of *Feml2*. Furthermore, we used the CAST and B6 genome sequences to identify genes within *Feml2* potential impact by coding variation. Consistent with the high polymorphism rate between CAST and B6 (Keane *et al.* 2011), 37 of the 69 *Feml2* genes contained coding variants predicted to potentially impact protein function (Table 2). Additionally, we hypothesized that noncoding variants between CAST and B6 could be driving expression differences between the two strains, and that this change in expression could be responsible for the reduced femur length in CAST mice. Using RNA-seq data from tibial growth plates of CAST × B6 F1s, we identified six of the 69 genes in the *Feml2* region that are preferentially expressed from one parental allele.

Of the genes influenced by ASE, we note two that have the potential to be driving the observed femur length phenotype based on their known biological function. *Larp6*, also known as Acheron, is an RNA-binding protein that is specific to collagen mRNAs (Stefanovic *et al.* 2014). *Larp6* regulates the translation of type I collagen subunits through sequence-specific binding to conserved stem loops in the 5′-UTRs of collagen mRNAs (Zhang and Stefanovic 2016). Collagens, predominantly type I collagen, comprise 90% of the bone matrix and their processing is critical for proper skeletal development (Young 2003). It has been observed that overexpression of *Larp6* blocks ribosomal loading onto collagen mRNAs, reducing translation of collagen and bone matrix formation (Cai *et al.* 2010). In CAST × B6 F1s, the expression of *Larp6* was higher from the CAST allele, potentially consistent with shorter femurs in HG9 mice. *Adpgk*, also known as ADP-dependent glucokinase, catalyzes the phosphorylation of d-glucose to D-glucose-6-phosphate using ADP as the phosphate donor (Richter *et al.* 2012). Knockouts of *Adpgk* result in short stature and slender bones (Skarnes *et al.* 2011). In CAST × B6 F1s, the expression of *Adpgk* was lower from the CAST allele, consistent with shorter and more slender femurs in HG9 mice. The ASE of these genes suggests that they may be involved in the regulation of femur length.

We identified the human syntenic region for *Feml2*, and found that it contained a number of associations for human height identified through GWAS. Human height is partly influenced by long bone length (Lui *et al.* 2016), and this well-powered GWAS, conducted by Wood *et al.* (2014), provides a robust collection of loci associated with human height to compare with the *Feml2* region’s influence on mouse long bone length. This human region appears to be a hotspot for associations with height, further implicating the murine region as a region influencing femur length and, more generally, skeletal dimension (Figure 3). Further characterization of the genes in the mouse *Feml2* region is potentially applicable to the study of human height.

In conclusion, this study identified *Feml2*, a region of the murine genome influencing femur length, and identified genes with coding and regulatory alterations, a subset of which may be responsible for the effects of *Feml2*. Additionally, comparisons with its human syntenic region support the notion that *Feml2* may contain multiple polymorphic genes, which in aggregate are responsible for its effect on bone geometry. Further characterization of these candidate genes and the identification of the mechanism by which they alter femur dimension will aid in the study of bone geometry and bone strength.

## Supplementary Material

Supplemental material is available online at www.g3journal.org/lookup/suppl/doi:10.1534/g3.116.037424/-/DC1.
