# Supplementary material for: Genetic Dissection of a QTL Affecting Bone Geometry
Source: G3 (Bethesda). 2017 Jan 11;7(3):865–70. doi: 10.1534/g3.116.037424 (PMC5345717; doi:10.1534/g3.116.037424)
Supplement: Supplementary file 1 [file 865TableS1.docx]

Table S1: List of potentially high-impact CAST/EiJ variants within Feml2 genes. * expressed in mouse growth plated as determined by RNA sequencing. (.xlsx, 13 KB)

Available for download as a .xlsx file at:

http://www.g3journal.org/lookup/suppl/doi:10.1534/g3.116.037424/-/DC1/TableS1.xlsx
