# Supplementary material for: Genetic Dissection of a QTL Affecting Bone Geometry
Source: G3 (Bethesda). 2017 Jan 11;7(3):865–70. doi: 10.1534/g3.116.037424 (PMC5345717; doi:10.1534/g3.116.037424)
Supplement: Supplementary file 4 [file 865FileS1.docx]

File S1. QTL mapping information, including mouse IDs, phenotype information, and SNP marker identifiers, locations, and genotypes. (.csv, 131 KB)

Available for download as a .csv file at:

http://www.g3journal.org/lookup/suppl/doi:10.1534/g3.116.037424/-/DC1/FileS1.csv
