# Supplementary material for: Genetic Dissection of a QTL Affecting Bone Geometry
Source: G3 (Bethesda). 2017 Jan 11;7(3):865–70. doi: 10.1534/g3.116.037424 (PMC5345717; doi:10.1534/g3.116.037424)
Supplement: Supplementary file 6 [file 865FileS2.docx]

File S2: README file that contains detailed information describing the phenotype abbreviations included in File S1. (.csv, 1 KB)

Available for download as a .csv file at:

http://www.g3journal.org/lookup/suppl/doi:10.1534/g3.116.037424/-/DC1/FileS2.csv
